# A Wearable Gait Phase Detection System Based on Force Myography Techniques

**DOI:** 10.3390/s18041279

**Published:** 2018-04-21

**Authors:** Xianta Jiang, Kelvin H.T. Chu, Mahta Khoshnam, Carlo Menon

**Affiliations:** MENRVA lab, Schools of Mechatronic Systems and Engineering Science, Simon Fraser University, Metro Vancouver, BC V5A 1S6, Canada; xiantaj@sfu.ca (X.J.); khchu@sfu.ca (K.H.T.C.); mkhoshna@sfu.ca (M.K.)

**Keywords:** FSR band, force sensors, gait phase, gait recognition

## Abstract

(1) Background: Quantitative evaluation of gait parameters can provide useful information for constructing individuals’ gait profile, diagnosing gait abnormalities, and better planning of rehabilitation schemes to restore normal gait pattern. Objective determination of gait phases in a gait cycle is a key requirement in gait analysis applications; (2) Methods: In this study, the feasibility of using a force myography-based technique for a wearable gait phase detection system is explored. In this regard, a force myography band is developed and tested with nine participants walking on a treadmill. The collected force myography data are first examined sample-by-sample and classified into four phases using Linear Discriminant Analysis. The gait phase events are then detected from these classified samples using a set of supervisory rules; (3) Results: The results show that the force myography band can correctly detect more than 99.9% of gait phases with zero insertions and only four deletions over 12,965 gait phase segments. The average temporal error of gait phase detection is 55.2 ms, which translates into 2.1% error with respect to the corresponding labelled stride duration; (4) Conclusions: This proof-of-concept study demonstrates the feasibility of force myography techniques as viable solutions in developing wearable gait phase detection systems.

## 1. Introduction

Walking is one of the basic activities of humans in everyday life and gait analysis provides a systematic and quantitative means to assess human locomotion [1,2]. Gait analysis has a wide range of applications spanning from sports performance analysis [3] to fall detection [4] and clinical monitoring of gait [5,6,7,8]. The sequences for walking are interpreted as gait cycles and are characterized by two main phases: the stance phase and the swing phase. Depending on the application, a more detailed breakdown of gait cycles might be required and in such cases, four, six or even eight different phases have been considered [1,9]. Automatic detection of gait phases can be used in distinguishing walking styles [10], detecting foot-drop [11,12], and improving the gait through nerve stimulation using functional electrical stimulation (FES) [12,13].

The majority of gait phase detection methods are derived based on the kinematic characteristics of foot movements that can be obtained using inertial sensors [12,13], insoles with built-in pressure sensors [14], or a combination of both [9]. The methods using inertial sensors can typically achieve a high accuracy at moderate to high walking speeds or in self-paced walking. However, their performance noticeably degrades at lower walking speeds, which is usually the pace for individuals with difficulty in walking [15,16].

Another well-established gait partitioning technique is using instrumented insoles that usually embed pressure sensors into one or two synchronized insoles [17,18]. Insole-based methods can achieve very high accuracy, are robust to walking speed, and have been used as gold standard [19]. However, this technique calls for customized footwear and cannot accommodate barefoot walking.

In this paper, we investigate the feasibility of using a force myography-based technique as the basis for automatic gait phase detection. Force myography (FMG) has been primarily applied to monitoring activities of upper extremities [20,21,22,23,24,25]. However, its capabilities for monitoring activity in the lower extremities has been less frequently explored. The work presented herein builds upon our previous work that demonstrated that FMG can be effectively used to count steps in a controlled environment with an accuracy of 98.5% even at lower walking speeds [26,27]. In this study, we further explore the capabilities of the force myography technique. To this end, FMG signals are collected from nine healthy individuals walking on a treadmill. Considering the inefficacy of inertia sensors at lower walking speeds, we focus on slow-paced walking and collect the FMG data at three low speeds (1–2 km/h). The performance of the FMG band in detecting gait phase events is evaluated in terms of sample-based detection accuracy, phase-based accuracy, and temporal accuracy of gait phase transition times. A high gait phase detection accuracy of over 99.9% and short temporal error of 2.1% have been achieved. The results of this study demonstrate the effectiveness of the proposed technique in detecting gait phases.

## 2. Materials and Methods

### 2.1. Experimental Setup

**Figure 1:**
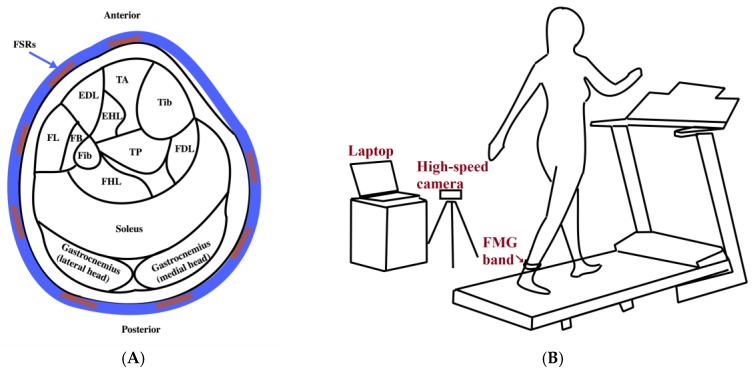

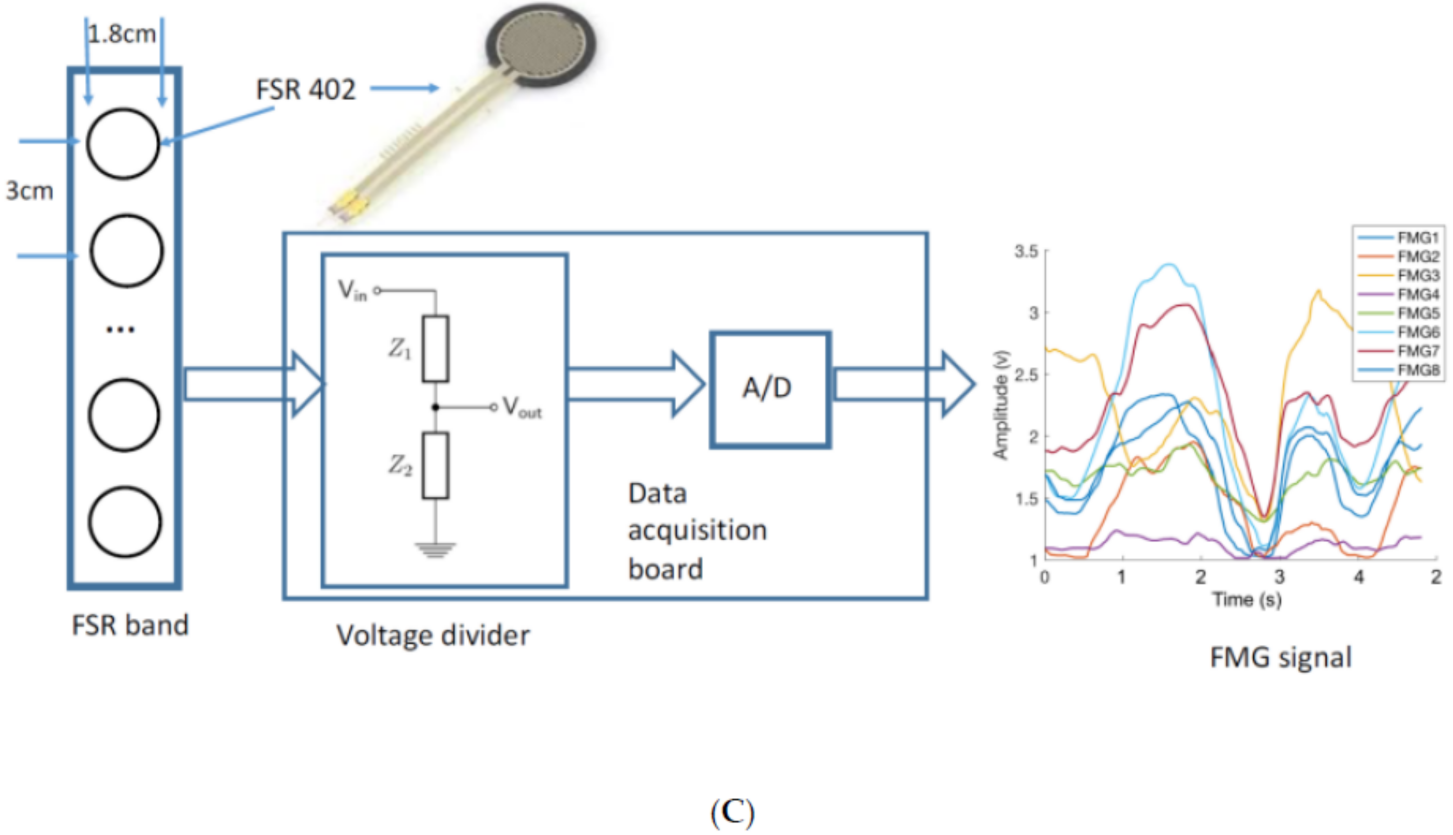
The experimental setup: (**A**) Schematic of the cross section of the ankle showing the main muscles and the location of force-sensing resistor (FSR) sensors (TA: Tibialias Anterior, EDL: Extensor Digitorum Longus, EHL: Extensor Hallucis Longus, Tib: Tibia, FL: Fibularis Longus, FB: Fibularis Brevis, Fib: Fibula, TP: Tibialis Posterior, FDL: Flexor Digitorum Longus, FHL: Flexor Hallucis Longus); (**B**) The force myography (FMG) ankle band is worn on the ankle of the dominant foot while the participant walks on a treadmill with a high speed camera recording the trial session; (**C**) The acquisition and conversion of the FMG signals.

The experimental setup used for data collection has three main parts: an FMG ankle band, a treadmill, and a high-speed camera (Figure 1). The FMG band consists of an array of eight force-sensing resistors (FSRs) (FSR 402, Interlink Electronics, Inc., Los Angeles, CA, USA) which are placed at equispaced points located at 3 cm from one another from the center of the FSR sensor along a 30-cm-length thin fabric sheet. Another piece of fabric is affixed to the inner layer of the band to have the wires covered. Velcro tape at the band ends ensures a snug fit around the ankle. Data from sensors are collected using a data acquisition board (NI USB-6008, National Instruments Inc., Austin, TX, USA) at a sampling frequency of 500 Hz. The illustration of the signal acquisition and conversion is shown in Figure 1C. The camera (Fastec Imaging Corporation, San Diego, CA, USA) is positioned to face the participant’s left sagittal side and to provide images with a resolution of 640 × 480 at 125 frames per second. These images provide the reference for the actual gait phase at each time instant.

The codes and interface required for data collection are created in LabVIEW. A change in the color of a radio button on the designed interface indicates the start of the data collection process and is used to synchronize the data collected from the band with acquired images. Machine learning algorithms and codes for data analysis are implemented using Statistics and Machine Learning Toolbox™ in MATLAB R2017b (The MathWorks, Inc., Natick, MA, USA).

### 2.2. Participants

Nine healthy volunteers (five males and four females) consented to participate in the study. The Office of Research Ethics at Simon Fraser University approved the study protocol (Study Number 2014s0590). The average age of participants was 26 ± 7 years and the average weight was 72.2 ± 15.3 kg. All participants self-reported full (100%) functionality of lower extremities. The score of the functionality reported ranging from 0 to 100%.

### 2.3. Protocol and Procedure

The study focused on slow-paced walking at three slow speeds: Speed1 = 1 km/h, Speed2 = 1.5 km/h, and Speed3 = 2 km/h. These values were determined based on the specifications of the available treadmill. The FMG band was donned on the participant’s dominant leg about 5 cm above the ankle and was strapped with sufficient but comfortable tightness. Since there are no muscles in the area around the Tibia, the band was placed around the ankle such that the Velcro ends covered this area and the FSR sensors were in contact with active muscles. The participants were asked to first complete a two-minute practice trial at Speed3 to familiarize themselves with the treadmill and the feel of the band around their ankle. The operator also asked the participants about the comfort of the FMG ankle band to readjust it if required. After completing the practice trial and a rest period of 2 min, the participants were asked to walk at one of the three speeds for 60 s. The subjects completed five trials at each walking speed in a randomized order of speeds. A two-minute rest period was considered between each two consecutive trials. Each participant completed 15 trials administered in one two-hour session of data collection. In each trial, the data collection was started after the initial acceleration period of the treadmill. The FMG data and the reference videos were saved to be processed later.

### 2.4. Data Analysis

#### 2.4.1. Reference Gait Labels

**Figure 2:**
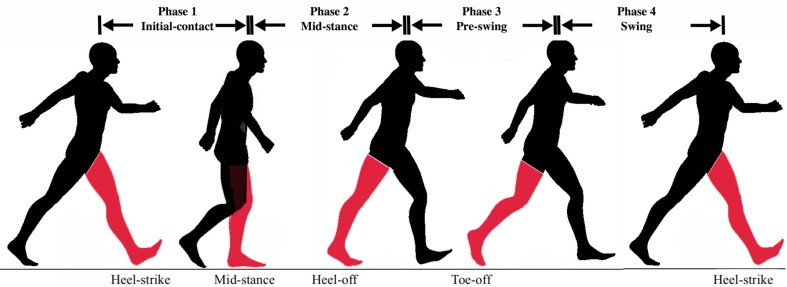
The gait phases illustration.

In this study, four gait phases are considered as shown in Figure 2: (1) Initial-Contact (IC), (2) Mid-Stance (MSt), (3) Pre-Swing (PS), (4) Swing (Sw) [9]. The Initial-Contact starts when the working heel strikes the ground until the opposite foot’s toe leaves the ground (single limb support). The Mid-Stance phase starts immediately after that and continues until the heel starts to break from the ground. The Pre-Swing phase starts from heel-off and ends with toe-off. The Swing phase is then defined as the interval between toe-off and heel-strike which marks the beginning of the next cycle. To determine the actual gait phase at each time instant, two independent observers examined the captured videos frame-by-frame and noted down the timestamp for each of the abovementioned events. The reference gait labels for each time interval are then created by averaging the results from both raters.

#### 2.4.2. Sample-Based Phase Classification

Linear Discriminant Analysis (LDA) is employed to determine the gait phase to which each data point belongs. The efficacy of LDA algorithm in achieving good classification results using myography signals has been previously demonstrated [23,24,28]. The LDA algorithm is readily available in MALTAB Statistics and Machine Learning Toolbox. The discriminant type of LDA in this study is pseudolinear and the default value of the linear coefficient threshold is set to 0.

Prior to applying the classification algorithm, feature extraction is applied using a 125 ms sliding window with 93 ms overlap. Therefore, the signal data are downsampled from 500 Hz to 125 Hz after feature extraction. A total of 14 features are extracted from the data: root-mean-square (RMS), sum of absolute value (SAV), mean absolute deviation (MAV), variance (VAR), wave length (WL), slope sign changes (SSC), and simple square integral (SSI), mean wavelet with db7 (db7), difference absolute standard deviation value (DASDV), average amplitude change (AAC), log detector (LD), linear fit (LF), and parabolic fit (PF) [25,29]. To reduce the computational cost of the algorithm while preserving its performance, the extracted features are normalized using the training data:

(1) $$X\_norm=\frac{X\_ori-\min\left(X\_train\right)}{\max\left(X\_train\right)-\min\left(X\_train\right)}$$

where X_ori, X_train, and X_norm represent the original data to be normalized, the training data, and the normalized data, respectively.

#### 2.4.3. Detection of Gait Phase Events

Gait phase event detection refers to determining the time of phase transitions based on the data samples classified as described in Section 2.4.2. In this study, we adapt the approach used in [13] to correct the gait phase corresponding to each data sample according to a set of rules. In this present method, the phase of each sample is compared to its previous one to check if a phase transition has occurred. Questionable phase transitions are those in which phases do not follow the correct sequence of the four phases as defined in Section 2.4.1. Skipping a phase or several phases or going back to any of the previous phases are instances of such incorrect event detections [13]. If the detected phase transition is questionable, next transitions will be compared to the latest correctly detected phase transition until transitioning to the next acceptable phase is detected. The segment consisted of these questionable transitions is called the recovery period. A set of rules is defined herein to correct questionable phase transitions based on the length and order of the phases in the recovery period, for example:

- If the duration of the recovery period is shorter than a certain length, e.g., half of the average of the detected phase duration, the transitions in this period are discarded.
- If the duration of the recovery period is longer than a certain length, e.g., the average gait cycle duration, this period is considered as a cycle. In the case in which this rule results in less than four phases in a gait cycle, it means that a phase transition is missed. Therefore, the phase corresponding to the recovery period is split in two to account for the missed transition.

#### 2.4.4. Performance Evaluation

The performance of the proposed gait phase detection method is quantitatively assessed based on three different measures of accuracy: sample-based, gait phase-based, and temporal error. The sample-based accuracy shows the level of agreement between the reference label of each data sample (Section 2.4.1) and its predicted label determined by the machine learning model:

(2) $$Sample\;based\;accuracy=\frac{\mathrm{correctly}\;\mathrm{classified}\;\mathrm{samples}}{\mathrm{total}\;\mathrm{number}\;\mathrm{of}\;\mathrm{samples}}\times 100\%$$

To validate the implemented machine learning algorithm for the sample-based accuracy, a cross-trial validation method is used: One of the five trials at each walking speed is chosen as the testing data and the remaining trials at that speed are assigned to the training dataset. This process is repeated until all trials have been considered once as testing data. The accuracy is then calculated by averaging the accuracies obtained from all five same-speed trials. The above cross-trial validation is performed for each subject.

In contrast to the sample-based accuracy, the so-called phase-based accuracy is calculated based on the gait events detected by the method as described in Section 2.4.3. According to the detected event moments compared to true labeled gait events, phase insertions and deletions are determined. A phase insertion is considered when a predicted gait phase segment does not match to its corresponding reference phase segment within a certain time window, e.g., 33% of the average duration of the true labelled gait cycle, which is normally longer than a phase duration. A phase deletion is defined when a true labelled phase misses its corresponding predicted phase segment. The gait phase-based accuracy is assessed by the percentage of the number of phases inserted and deleted compared to the number of correctly detected phases.

The absolute temporal error of the gait event detection is measured by the absolute time difference between the detected phase transition time point and its corresponding true label phase transition time point. A relative temporal error is also calculated by dividing the absolute time difference to the mean duration of corresponding strides.

One-way analysis of variance (ANOVA) is applied to examine how the walking speed affects the sample-based accuracy. Two-way ANOVA is applied to characterize how the walking speed and type of phase affect the temporal accuracy measurement. Post Hoc pair comparison (Tukey HSD) is conducted to determine if there is any significant effect of the variables on the accuracy. The significance level is set to *p*-value = 0.05. The ANOVA assumes that the input variables are normally distributed. The Kolmogorov–Smirnov test is used to verify this assumption for the obtained values before ANOVA is conducted. The ANOVA and Kolmogorov-Smirnov test were done using Statistics and Machine Learning Toolbox™ provided by MATLAB R2017b.

## 3. Results

Each participant completed 15 trials (three speeds in each of the five trials). Considering the sampling rate and the duration of each trial, 30,000 FMG data points were collected from each subject and a total of 4,050,000 samples were collected across 135 recorded trials. After feature extraction, the signal for each trial was downsampled to 7500 samples (Section 2.4.2). Taking into account that there were 8 signals in accordance with the 8 FSRs in the ankle band and that 14 features were selected from each signal, there were 112 features for each of the 7500 data samples of each speed-trial.

**Figure 3:**
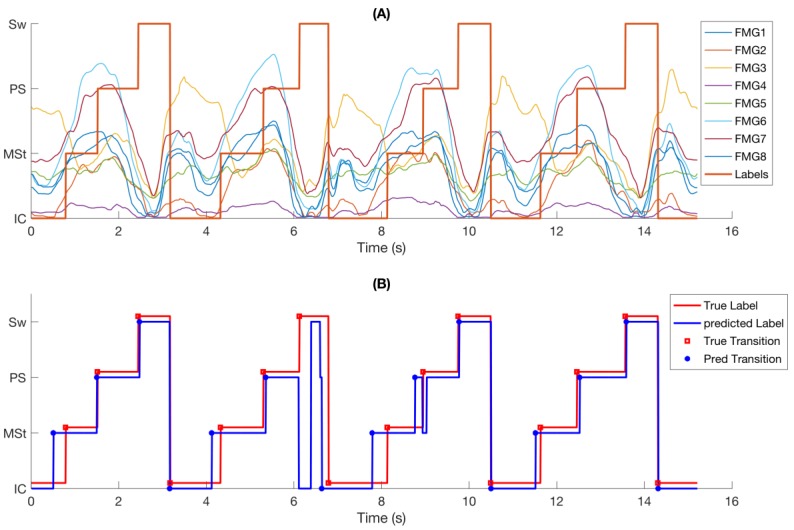
Example FMG raw signals for a segment of 4 gait cycles (**A**) raw FMG signals overlayed with reference labels (red solid line) determined from the recorded video (from subject 5, trial 5, Speed1); (**B**) predicted phase labels (blue solid line) using LDA as well as the true phase transitions and predicted phase transitions after applying supervisory rules. A deletion happens at about 6.1 s.

FMG signals demonstrated consistent response to changes in gait phases (Figure 3A). Similar patterns were observed in FMG signals from one gait cycle to another, but the trend and amplitude of signals were different for each phase within each gait cycle. It is observed that the FMG signals had an upward trend from Initial-Contact to Pre-Swing and their amplitude increased to a peak value before it quickly dropped at the end of Pre-Swing.

Figure 3B shows the same data as in Figure 3A with reference labels, predicted phase class labels, reference phase transitions, and predicted phase transitions. It is observed that most of the transitions were correctly predicted with little temporal deviation from the true labels. A short incorrectly detected transition at 8.4 s has been corrected by the supervisory rules (Section 2.4.3). However, a longer questionable transition of about 200 ms at 6.1 s could not be corrected and was considered as a phase deletion.

### 3.1. Intra-Rater Agreement

As explained in Section 2.4.1, the reference gait labels were created by asking two independent observers to note down the frame of video indicating the occurrence of each of the considered gait events. To evaluate the degree of agreement between these observers in extracting the video frames, the intraclass correlation coefficient (ICC) was obtained using an average measure, absolute-agreement, two-way mixed-effects model [30]. Considering all 135 trials, ICC was 0.9744 with 95% confidence interval = 0.9743 – 0.9746, indicating excellent intra-rater agreement in obtaining reference labels.

### 3.2. Sample-Based Accuracy

**Figure 4:**
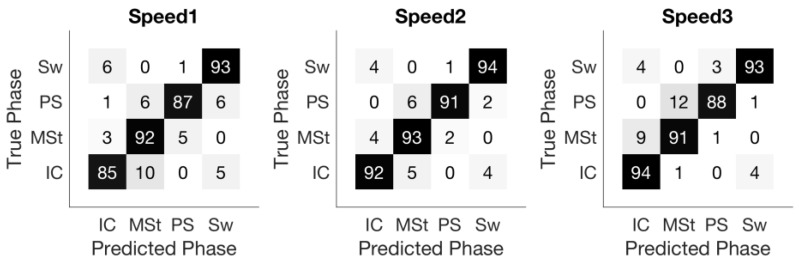
Confusion matrix of sample-based accuracy in different speeds. Misclassification occurs mostly during phase transitions.

The overall sample-based accuracy was 91.3% ± 3.3% across nine subjects, with speed related accuracies of 89.5% ± 2.5%, 92.9% ± 1.2%, and 91.4% ± 1.3% for the three speeds, respectively. Figure 4 shows the confusion matrix of the sample-based prediction. Not surprisingly, misclassification mostly occurred in transitioning between two consecutive gait phases. For instance, in the matrix corresponding to Speed1, 2% and 4% of Mid-Stance samples were misclassified as Initial-Contact and Pre-Swing, respectively, but no Mid-Stance samples were classified as Swing.

### 3.3. Phase-Based Accuracy and Temporal Error

A very high phase-based accuracy was achieved. For all three speeds across nine subjects (12,965 phase segments), four phase deletions were required. No instance of phase insertion was observed.

**Figure 5:**
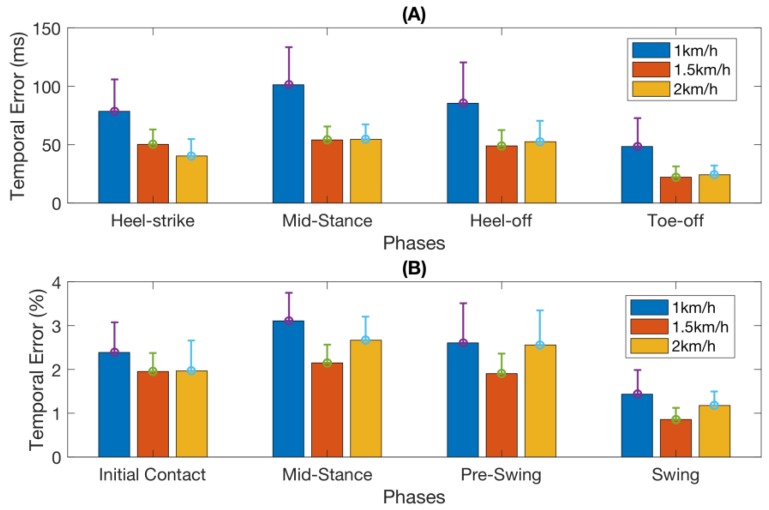
Mean temporal error for each phase across nine subjects spanned by three speeds. (**A**) The absolute temporal error of the phase transitions; (**B**) Relative temporal error. The error bars stand for 1 standard deviation.

Table 1 summarizes the mean absolute as well as relative temporal errors (as defined in Section 2.4.4) in detecting each of the four gait events across all subjects and all walking speeds. This information is also illustrated in Figure 5.

### 3.4. ANOVA Results

The Kolmogorov–Smirnov test confirmed that the derived accuracy and temporal error data were normally distributed, and hence suitable for ANOVA. The one-way ANOVA showed a significant effect of walking speed on sample-based accuracy (F_2,26_ = 6.83, *p* < 0.005). The Post Hoc test (Tukey HSD) revealed the sample-based accuracy of Speed1 (the lowest speed) is significantly lower (*p* < 0.005) than that of Speed2, but there was no other significant difference between Speed1 and Speed3, and between Speed2 and Speed3.

The two-way ANOVA data analysis revealed significant effects of both the gait phase (F_2,107_ = 16.22, *p* < 0.00001) and the walking speed (F_2,107_ = 31.2, *p* < 0.00001) on the absolute temporal error, but there was no significant interaction effect between the walking speed and the gait phase. The Tukey HSD on the effect of the gait phase showed that the temporal error of phase 4 (Swing) is significantly lower than that of other phases (*p* < 0.0005), but there was no significant difference between the other three phases (Phases 1–3). The Tukey HSD on the effect of walking speed showed that the temporal error at Speed1 was significantly higher than that at Speed2 (*p* < 0.00001) and Speed3 (*p* < 0.00001), but there was no significant difference between those at Speed2 and at Speed3.

The two-way ANOVA data analysis also reported significant effects of both the gait phase (F_2,107_ = 23.76, *p* < 0.00001) and walking speed (F_2,107_ = 6, *p* < 0.005) on relative temporal error, but there was no significant interaction effect between the walking speed and the gait phase. The Tukey HSD on the effect of the gait phase showed that the temporal error of phase 4 (Swing) was significantly lower than those of other phases (*p* < 0.00001), but there was no significant difference between the other three phases (Phases 1–3). The Tukey HSD on the effect of walking speed showed that the temporal error at Speed1 was significantly higher than that at Speed2 (*p* < 0.005) and there was a marginal significant difference between Speed1 and Speed3 (*p* = 0.079), but there was no significant difference between those of Speed2 and Speed3.

## 4. Discussion

The contraction and relaxation of the extensor and flexor muscles at the ankle position [31,32]. during gait phases alters the pressure distribution resulting in distinctive FMG patterns sensed by the FSR strap. Therefore, the FMG data can be used to obtain information about an individual’s gait patterns. Using the FMG data collected from an in-house developed ankle band, more than 99.9% of gait phase transitions were correctly detected: only four phase deletions were occurred over a total of 12,965 gait phases. The average absolute and relative temporal errors were 55 ms and 2.1%, respectively. The performance of this gait event detection system, including insertion and deletion rates and relative temporal error, is comparable to those reported in other studies as summarized in Table 2.

From Table 2, the absolute temporal error of the method proposed herein (55.2 ms) is slightly higher than that of other methods. However, when the walking speed, i.e., the duration of average gait cycles, is taken into account, the relative temporal error of our system (2.1%) is among the best. Moreover, the phase-based accuracy (insertion and deletion rates) is comparable to that achieved using insole FRSs [9] with the added benefit that the proposed FMG band does not require instrumented footwear and can be used in barefoot walking as well. The overall sample-based accuracy was about 91%, which is slightly lower than that reported in literature. However, the majority of misclassified data samples occurred near phase transitions (Figure 3B). Considering that these transition intervals are typically not long enough to cause misclassification of the gait phase segment and also that the rule-based supervisory correction handles any short misclassification during non-transition periods (Figure 3B), it is not surprising that a very high phase-based accuracy was achieved.

The confusion matrix of sample-based accuracy as shown in Figure 4 confirms the above observation. Almost all misclassified samples happen between adjacent phases and contribute mainly to the temporal error of the gait phase detection.

Walking speed affected the accuracy significantly: lower sample-based accuracy and larger temporal error were achieved at the lowest speed (1 km/h) (Section 3.2 and Figure 4). ANOVA on the walking speed revealed its significant effect on the sample-based accuracy and the absolute temporal error. This performance degradation might partially be due to the increase in signal variations at this almost impractical walking speed. However, the FMG band still achieved a decent phase-based accuracy with only two deletions at Speed1 across all trials performed by all subjects.

The significant effect of walking speed was reduced when considering the relative temporal error (Figure 3B). ANOVA shows the relative temporal error of Speed1 is only significantly higher than Speed2.

**Figure 6:**
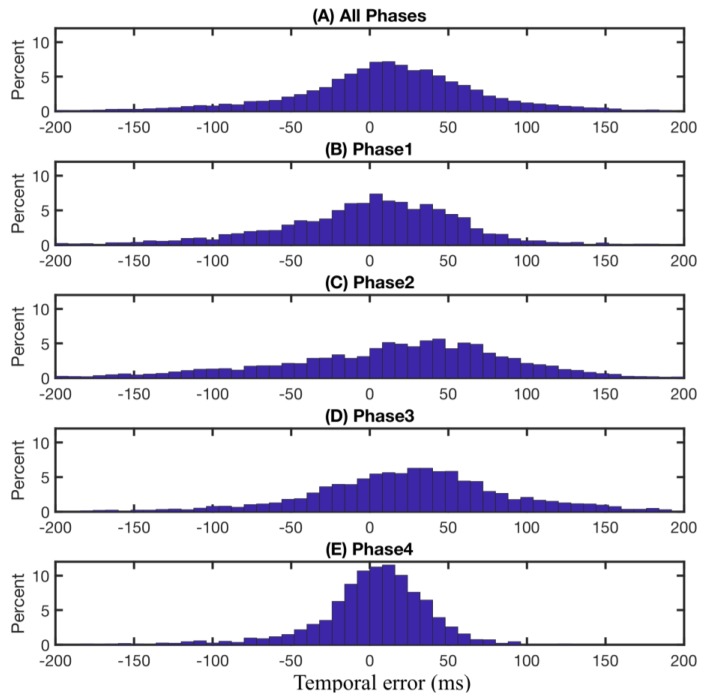
Time difference (temporal error) between detected gait phase events and manually annotated gait events. Positiveness of the durations means the predicted events proceed the corresponding observed events. (**A**) overall temporal error considering all four gait phases across nine subjects and all three speeds. (**B**–**E**) temporal errors for each gait phase across all nine subjects and all three speeds.

In terms of time delay, overall, the FMG-based phase partitioning system detects the gait phase transitions slightly earlier than those observed from recorded video, as shown in Figure 6A. This early detection effect is especially significant for the detection of transitions from phase 1 to phase 2 (Initial-Contact to Mid-Stance) and from phase 2 to phase 3 (heel breaks up the ground), as shown in Figure 6C,D. This result is expected since these two transitions involve more active muscular contractions around the ankle area [32]. During the loading response (after Initial-Contact) to mid-stance, the ankle dorsiflexors contract to control the flexion movement. During the transition from phase 2 to phase 3, the toe flexors and tibialis posterior contract to break up the heel from the ground and push the foot against the ground to move the body forward. On the contrary, the transitions from phase 4 to phase 1 (heel strikes the ground) and phase 3 to phase 4 (Toe-off) do not engage the muscles around the ankle area as much. For example, at the beginning of phase 4, hip flexors contract to propel the limb and at the end of phase 4, hamstring muscles are mainly involved to decelerate the forward motion of thigh. Both of these muscle groups are not located at the ankle area and thus, cannot be detected by the ankle band. This point is confirmed by observing the variations of FMG signals shown in Figure 3A. The increasing trend of amplitude of most channels of FMG signals starts later in phase 1 and continues to early in phase 3, but the signal amplitude decreases after that.

## 5. Limitations and Future Work

This preliminary study explored using an FMG band for gait phase detection. The study was conducted in a controlled environment where the participants were asked to walk on a treadmill. Moreover, the participants wore the band throughout the entire data collection session. However, it is expected that the signals would change if the band is taken off between trials since the position of the band around the ankle and its tightness might change. Further studies should consider testing in more relaxed environments and investigating the robustness of FMG readings.

Towards developing a non-obtrusive method to characterize slow-paced walking, this study focused on relatively slow walking speeds at which the performance of the main stream wearable devices degrades. Future studies will consider evaluating the proposed technique at a wider range of walking speeds and integrating an IMU in the ankle band to enhance its performance.

In this study, the data from the ankle band was collected and transferred to the computer via a data acquisition card. Therefore, although the designed ankle band is light and unobtrusive, the data transmission is not wireless, which means this first prototype is not a stand-alone wearable device. In future designs, wireless data transmission technologies, such as Bluetooth, will be considered.

The reference gait labels in this study were obtained by manually labelling the recorded video, which is a tedious task. Motion tracker systems or in-sole pressure sensors are examples of methods that can automatically generate reference labels and therefore, allow for increasing the number of subjects or the number of trials without overburdening the labelling task. Future research should also explore unsupervised learning methods to improve the usability of the FMG band for gait phase detection during daily activities by discarding the need for reference labels to train the model.

## 6. Conclusions

This paper presented a wearable gait phase detection system based on force myography techniques. An array of 8 pressure sensors were embedded into an ankle band to monitor foot movements during walking. Nine participants were asked to wear the band and to walk on a treadmill at different speeds. An LDA classifier was employed to implement a sample-based phase classification enhanced further with a rule-base supervisory phase transition correction. It was shown that the system achieves a gait phase-based detection accuracy higher than 99.9% at all studied speeds and the temporal error with respect to the stride duration is at 2.1%. The results of this study suggest the feasibility of using an FMG-based ankle band to detect gait phases during slow-paced walking.

## Figures and Tables

**Table 1:** Mean temporal error obtained for each of the four gait events across all subjects and all walking speeds.

Measure of Temporal Error	Phases			
	1. Initial-Contact	2. Mid-Stance	3. Pre-Swing	4. Swing
Absolute temporal error (ms)	56.3 ± 15.5	70.1 ± 15.6	62.3 ± 19.2	31.6 ± 13.2
Relative temporal error (%)	2.1 ± 0.5	2.6 ± 0.4	2.4 ± 0.6	1.2 ± 0.3

**Table 2:** Performance of several gait phase detection systems reported in the literature. The number of gait phases considered in these papers is four. The relative temporal error is calculated by dividing the absolute temporal error to the stride duration.

Paper	Sensors	Healthy Subject #	Sample Accuracy or (TPR, TNR)	Phase accuracy (Deletions, Insertions)	Absolute Temporal Error (ms)	Relative Temporal Error (%) (Stride Duration, Speed)
Rueterbories et al. [33]	Acceler-ometers	10	(>96%, >91%)	(15.8%, N/A)	50.2	3.2% (1.55 s, 70 steps/min)
Mannini et al. [34]	Gyro	4	(95%, 95%)	(0%, 12%)	35	3.0% (1.15 s, 3–6 km/h)
Taborri et al. 2014 [35]	IMU	10	(>95%, >95%)	N/A	N/A	N/A (1.60 s, 1.8–5.4 km/h)
Mannini et al. [10]	Gyro	6	(95%, 98%)	(0%, 4.3%)	< 20	N/A (N/A, 3–7 km/h)
Mannini et al. [36]	Gyro	9	N/A	(0%, 0.45%)	45–35	3.7% (1.09 s, 4.8 km/h)
Pappas et al. [9]	Gyro, insole FSRs	10	N/A	(0%, 0%)	53.8	4.5% (1.2 s, 3 km/h)
The present paper	FMG on ankle	9	91.2%	(0.03%, 0%)	55.2	2.1% (2.60 s, 1–2 km/h)
